# Psychiatric Illnesses as a Cause of Early Retirement

**DOI:** 10.1192/j.eurpsy.2026.11837

**Published:** 2026-07-31

**Authors:** W. Ayed, G. Bahri, Y. Yaich, G. Amri, K. Grissa, Y. Tebourbi, S. Chemingui, D. Brahim, M. Bani, R. Ghachem, N. Ladhari

**Affiliations:** 1 Occupational Medicine Department, Charles Nicolle Hospital, Tunis; 2 Department B, Razi hospital, Mannouba, Tunisia

## Abstract

**Introduction:**

Psychiatric disorders can be severely disabling, limiting daily activities, professional functioning, and long-term work participation

**Objectives:**

The aim of the study was to identify the socio-professional characteristics of patients granted disability benefits or early retirement due to premature wear and tear on the body caused by psychiatric disorders.

**Methods:**

A descriptive, retrospective study was conducted on medical records of patients who were referred to the occupational health department at Charles Nicolle University hospital in Tunis for early retirement between 1 January 2017 and 31 March 2025.

**Results:**

Of 146 applications for early retirement, 31% (n=27) were related to psychiatric disorders. The mean age was 54±3 years with a predominance of women (56%). The mean professionnal seniority was 24 ± 7 years. The most represented occupational sectors were health (37%) and textiles (18%). The most represented occupations were manual labourers (52%). The psychiatric disorders observed were: anxiety-depression disorders (80%) and schizophrenia (20%). Early retirement was granted due to premature wear and tear on the body in 85% of cases and disability in 15% of cases.

**Conclusions:**

Our study indicates that psychiatric disorders represent a major cause of early withdrawal from the working life, highlighting the need for improved prevention strategies and workplace accommodations to help patients manage and live with their condition

**Disclosure of Interest:**

None Declared

